# Implementing the right care in the right place at the right time for non-alcoholic fatty liver disease (NAFLD-RRR study): a study protocol for a community care pathway for people with type 2 diabetes

**DOI:** 10.1186/s12913-022-07808-7

**Published:** 2022-04-12

**Authors:** Lucy Gracen, Kelly L. Hayward, Melanie Aikebuse, Anthony Russell, James O’Beirne, Steven McPhail, Katharine M. Irvine, Suzanne Williams, Patricia C. Valery, Elizabeth E. Powell

**Affiliations:** 1 Centre for Liver Disease Research, Faculty of Medicine, Translational Research Institute, The University of Queensland, Level 5, West Wing, 37 Kent Street, Woolloongabba, Brisbane, QLD 4102 Australia; 2 Department of Gastroenterology and Hepatology, Princess Alexandra Hospital, Brisbane, QLD Australia; 3 Centre for Health Services Research, Faculty of Medicine, The University of Queensland, Woolloongabba, QLD Australia; 4 Department of Endocrinology, Princess Alexandra Hospital, Brisbane, QLD Australia; 5 Department of Gastroenterology and Hepatology, Sunshine Coast University Hospital, Birtinya, QLD Australia; 6 Australian Centre for Health Services Innovation School of Public Health, Institute of Health and Biomedical Innovation, Queensland University of Technology (QUT), Brisbane, QLD Australia; 7 Digital Health and Informatics, Metro South Health, Brisbane, QLD Australia; 8 Mater Research, Translational Research Institute, Brisbane, QLD Australia; 9 Inala Primary Care General Practice, Inala, QLD Australia; 10 QIMR Berghofer Medical Research Institute, Brisbane, QLD Australia

**Keywords:** Cirrhosis, Diabetes complications, Patient care, Risk assessment, Delivery of health care, Integrated

## Abstract

**Background:**

Non-alcoholic fatty liver disease (NAFLD) is an emerging epidemic that affects approximately half of all people with type 2 diabetes. Those with type 2 diabetes are a high-risk NAFLD subgroup because of their increased risk of clinically significant liver-related outcomes from NAFLD which include hepatocellular carcinoma, cirrhosis-related complications and liver disease mortality. They may benefit from early detection of disease as this would allow at risk patients to access hepatocellular carcinoma surveillance, emerging drug trials for NAFLD and specialist hepatology care prior to emergence of liver-related complications.

**Methods:**

This is a prospective cohort study aimed at incorporating and assessing a community care pathway for liver fibrosis screening into routine care for type 2 diabetes. Patients undergo a point of care assessment of hepatic steatosis and stiffness using FibroScan at the time of the routine diabetes appointment or when attending the clinic for blood tests in preparation for this appointment.

**Discussion:**

We propose that implementation of a community-based NAFLD diagnosis, risk-stratification, and referral pathway for people with type 2 diabetes is feasible, will provide earlier, targeted detection of advanced fibrosis, and reduce unnecessary referrals to hepatology outpatients for fibrosis risk assessment. Our study will provide important information about the feasibility of establishing a NAFLD pathway for people with type 2 diabetes in primary care. Ultimately, our findings will help direct spending and resource allocation for NAFLD in a high-risk population. Regular evaluation by stakeholders during implementation will help to create a reliable and sustainable community care pathway and establish a perpetual cycle of learning in primary care.

**Trial registration:**

ANZCTR, ACTRN12621000330842. Registered 23 March 2021.

**Supplementary Information:**

The online version contains supplementary material available at 10.1186/s12913-022-07808-7.

## Novelty statement

- There is an inconsistent approach to testing for and evaluating NAFLD in the community, which raises concerns that high-risk subgroups, including people with type 2 diabetes, are under-investigated and managed for advanced hepatic fibrosis.
- This study describes a method for implementing and evaluating a “real-world” referral pathway for the emerging epidemic of NAFLD.
- We have designed this community care pathway to be regularly evaluated by stakeholders during its implementation and through this review process to create a perpetual cycle of learning in primary care.

## Background

Non-alcoholic fatty liver disease (NAFLD), the liver component of a group of conditions associated with metabolic dysfunction, is present in 47–64% of people with type 2 diabetes (T2D) [1]. NAFLD is a heterogeneous disorder with variable rates of liver disease progression and clinical outcomes [2–4]. Although the leading causes of death are cardiovascular disease and extrahepatic malignancy, 5–10% of people with NAFLD develop complications of advanced liver disease over 10 to 20 years [5, 6]. Among people with T2D, up to one third (15–30%) are at risk of clinically significant fibrosis [7–9] and there is a more than 2-fold increased risk of cirrhosis-related complications and liver disease mortality [10]. As a result, there is increasing recognition that an assessment of NAFLD and the severity of liver disease needs to be incorporated into the routine care of patients with T2D [7, 9, 11].

Several peak bodies recommend identifying people with T2D or metabolic syndrome at risk of advanced chronic liver disease (CLD) [12–14]. In the absence of a structured approach to screening and risk stratification, the diagnosis of NAFLD in these high-risk people is inconsistent and often occurs incidentally, during investigation of other clinical concerns [15, 16]. These patients are more likely to have severe liver disease and hepatocellular carcinoma (HCC) at the time of diagnosis [17], which greatly limits available therapeutic options. In contrast, the majority of patients currently referred to hepatology clinics for evaluation of NAFLD do not have advanced fibrosis and could be appropriately managed in primary care with attention to cardiometabolic risk factors. At present there is limited data regarding how best to support the targeted detection of patients at highest risk of CLD among the immense number of people with T2D.

The most important prognostic marker for adverse liver outcomes and overall mortality is the presence of advanced fibrosis [5, 6, 18, 19], which can be assessed using combinations of non-invasive tests [19, 20]. Clinical judgement and liver enzymes are poor discriminators of NAFLD severity. In people with T2D, transient elastography can identify the subgroup of individuals with a high risk of advanced fibrosis. Screening studies in community [21] or hospital-based T2D clinics [22, 23] have shown that, using a liver stiffness measurement (LSM) threshold of ≥9.6 kPa, advanced fibrosis was confirmed in 40–50% of patients who received a liver biopsy [24, 25]. Using a 8.0 kPa cut-off to exclude advanced CLD has a sensitivity of 93% in NAFLD [26]. Transient elastography can be performed as a rapid point-of-care test and allows estimation of steatosis by controlled attenuation parameter (CAP) at the same time. Although clinical guidelines endorse the use of simple scores (NAFLD Fibrosis Score (NFS) and Fibrosis 4 Index (FIB-4)) for initial assessment of fibrosis, a substantial proportion (36–63%) of patients fall into an “indeterminate risk” category and require second-line assessment with transient elastography [8, 27].

To date, few studies have examined strategies to introduce routine assessment of fibrosis into T2D care pathways. We propose that implementation of a community-based NAFLD diagnosis, risk-stratification, and referral pathway for people with T2D is feasible, will provide earlier, targeted detection of advanced fibrosis, and reduce unnecessary referrals to hepatology outpatients for fibrosis risk assessment.

## Methods/design

### Overview

The non-alcoholic fatty liver disease: implementing the right care in the right place at the right time (NAFLD-RRR) study is a quasi-experimental study with a prospective intervention and historical control group. The intervention aims to incorporate a community care pathway for liver fibrosis screening into routine care for T2D. The study protocol (Version 6, 9th Feb 2022) includes in-depth phenotyping of participants including demographic details, metabolic co-morbidities, anthropometric measurements, alcohol consumption, smoking status, current medications and average daily physical activity. During a single assessment, liver steatosis and stiffness are measured using an EchoSens FibroScan Mini+ 430. The patient record is interrogated for the most recent haematological and biochemical parameters, lipid profile, and abdominal radiology. Patients are also asked for consent to collect health care usage data from a national Government agency, Services Australia. These data will be used to determine longitudinal health outcomes and facilitate an accurate and thorough analysis of health care usage and economic costs.

### Study aims

The primary aims of the study are to determine whether an integrated diagnostic and risk stratification pathway for people with T2D in primary care:

A1: increases the rate of detection of NAFLD and.

A2: increases the number of people identified with advanced fibrosis/cirrhosis.

The secondary aims are to determine whether the pathway:

A3: links people with NAFLD to the “right care” tailored to individual needs (referral of people with increased risk of advanced liver fibrosis to Hepatology clinics) and.

A4: increases the rate of surveillance for HCC among people with a diagnosis of cirrhosis.

We will also evaluate:

A5: the sustainability of the intervention in the current health care system in relation to resource use and costs, and.

A6: the utility of pathway-specific template letters to the General Practitioner (GP) and diabetes specialist that summarise the clinical findings and provide brief recommendations for management and follow-up.

### Hypotheses

Compared with patients receiving usual care, people with T2D using the NAFLD pathway are hypothesized to benefit in the following ways:

H1: increased identification of NAFLD.

H2: increased identification of people at increased risk of advanced fibrosis/cirrhosis.

H3: increased referral of people with ‘high-risk’ NAFLD to Hepatology clinics and reduced referrals of ‘low-risk’ NAFLD to Hepatology clinics.

H4: increased surveillance for HCC among people with a diagnosis of cirrhosis.

H5: resource use and net-costs of the new pathway for NAFLD diagnosis and risk stratification in the community will be sustainable relative to existing usual care.

H6: GP knowledge and confidence in the recognition, diagnosis, risk stratification and management of NAFLD in their practice population will improve.

### Design and setting

This clinical trial examines the implementation of a diagnostic and risk stratification pathway for NAFLD in people with T2D. Sequential participants will be recruited to the NAFLD-RRR pathway and followed for up to 10 years to monitor their health outcomes. The detection of patients with NAFLD ± advanced fibrosis and the surveillance for HCC among “high-risk” patients will be compared to patients receiving “usual care”.

Due to the high prevalence of NAFLD and advanced fibrosis in people with T2D, it is not appropriate to conduct a randomized study of screening and risk stratification versus no intervention. All people attending the community diabetes clinics will be offered participation in the study. Study outcomes will be compared to a cohort who received “usual care” at the community diabetes clinics in the 12-months prior to implementation of the NAFLD-RRR pathway. The medical records of “usual care” patients will be audited to determine the number of people with a recorded diagnosis of NAFLD, whether an assessment of liver disease severity was performed, and the number of people referred to a liver clinic.

The study will be conducted in 3 “Beacon” diabetes clinics in the Metro South hospital and health services district (HHS), where people who are referred by their GP for specialist management of T2D are offered specialised care in a primary care setting. The Beacon model for people with complex T2D is delivered through community-based general practices where GPs with a special interest (GPwSIs) and advanced training in diabetes work alongside an endocrinologist and diabetes nurse educator [28]. The model is an alternative to standard hospital outpatient review and is fully integrated, with primary care providers and specialists co-located to provide effective, cost-efficient and patient-centred care [29]. In this model the GPwSIs take their enhanced skills back to their routine practice, so they become a referral point and source of knowledge for colleagues, potentially reducing referrals to specialist clinics. Prior to developing and implementing the NAFLD-RRR pathway, diabetes clinic/community clinicians (*n* = 24) were invited to participate in focus group discussions to obtain information about preferences and strategies for learning, support and communication [30].

The Metro South HHS covers the south side of Brisbane and has a catchment area of 3860 km^2^. It provides healthcare to more than one million people, which is 23% of the state of Queensland’s population.

### Participants

#### Eligibility criteria

Eligible participants are (1) adults aged ≥18 years; (2) who are referred to a Beacon diabetes clinic in Metro South HHS for management of T2D.

#### Exclusion criteria

Individuals will be excluded if they are (1) unable to provide informed consent; (2) pregnant and/or; (3) have a diagnosis of advanced cardiac disease or another terminal illness, or known chronic liver disease and are undergoing follow-up with a Hepatology clinic.

### Participant recruitment

The diabetes clinic appointment databases will be prescreened for eligible participants who are scheduled for routine review by a diabetes clinician. Persons identified as eligible will be contacted via text message and then telephoned in the 2 weeks preceding their appointment to discuss the study and offer participation.

**Fig. 1:**
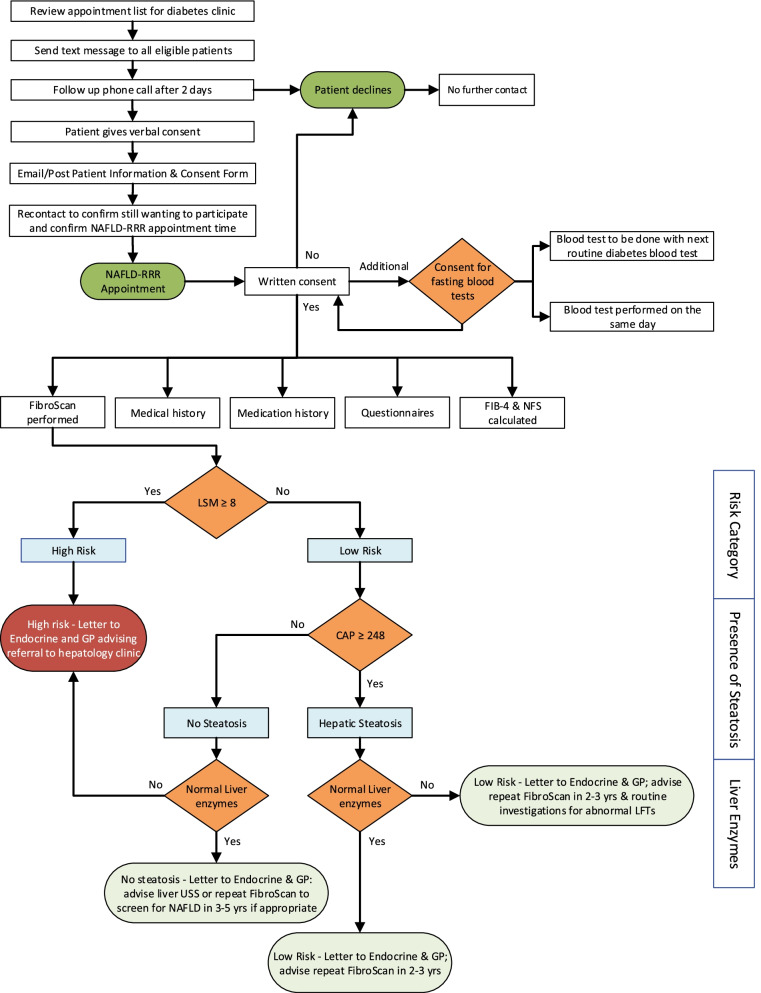
Flow diagram of initial recruitment strategy and patient flow through the NAFLD-RRR pathway. USS Ultrasound Scan; LSM Liver Stiffness Measurement; CAP Controlled Attenuation Parameter; LFTs Liver Function Tests; FIB-4 Fibrosis-4 Score; NFS Non-alcoholic Fatty Liver Disease Score; GP General Practitioner

Scheduled participants will be invited to be screened for NAFLD when they attend a Diabetes clinic appointment or when they have their fasting blood tests performed in preparation for their Diabetes clinic appointment, using FibroScan to assess steatosis (CAP score) and liver fibrosis (liver stiffness measurement, LSM) (Fig. 1).

### Intervention

#### FibroScan

Transient elastography (using FibroScan) has been extensively evaluated; a liver stiffness threshold of 6.5–7.9 kPa has approximately 90% sensitivity in excluding advanced (stage 3–4) fibrosis, whereas LSM > 12–15 kPa is typically present with cirrhosis [31–35]. Transient elastography will be performed by a trained clinician as a point-of-care test, concurrently with the diabetes clinic appointment or when the participant attends for a scheduled blood test, making it easier for participants to have the test performed.

To obtain accurate results from the FibroScan assessment participants will be advised to fast for 3 h before the scheduled time of examination. Clear guidance will be provided to participants regarding fasting and withholding of T2D medications. If the FibroScan appointment is prior to 10 am, participants will be advised to fast overnight and withhold T2D medications until after the examination. If the FibroScan appointment is 10 am or later, participants will be advised to have an early breakfast, and to take their usual T2D medications, and then to fast for 3 h prior to the scan. Participants will be advised to bring a snack and all medications to clinic so that as soon as the FibroScan is completed, they can eat and take their T2D medications as required.

In around 9–15% of examinations, a valid LSM cannot be obtained because a participant’s body habitus or skin to liver capsule depth prevents an accurate assessment [8, 36]. Should this occur, the need for further review of these participants will be determined by the diabetes and study clinicians based on the history, blood tests and imaging available.

#### Other clinical information

At the FibroScan assessment the study clinician will assess girth and document alcohol consumption using the short form AUDIT [37] to quantify alcohol misuse, based on 3 questions posed to patients about their consumption habits. A short medical history form (Table 1) will be completed to obtain information about the following: prior diagnosis, management and follow-up of fatty liver; prior hepatology review; year of diagnosis of T2D; presence of other cardiometabolic risk factors (obesity, hypertension, dyslipidaemia); presence of cardio- cerebro- or peripheral vascular disease, history of cancer; mental health; family history of fatty liver; medications; current employment status (full-time, part-time, unemployed, disability pension); sociodemographic data (e.g. marital status, education level, country of birth, place of residence).

**Table 1:** Clinical data collected at NAFLD-RRR appointment

Demographic information	
Age	
Ethnicity	
Language spoken at home	
Country of birth	
Sex	
Menopausal status – pre, peri, post	
Relationship status – single, married or de factor, divorced/separated, widowed	
Level of education – junior, senior high school, TAFE, university	
Employment status – full time, part time, unemployed, disability pension	
Medical history	
Year T2D diagnosed	
Cardiometabolic risk factors – obesity, hypertension, dyslipidaemia,	
Metabolic comorbidities – cardiovascular disease, atrial fibrillation, cerebro- and/or peripheral vascular disease, obstructive sleep apnoea, chronic kidney disease, gout	
History of malignancy – primary cancer type and year diagnosed	
Patient-reported NAFLD	
Previous diagnosis of NAFLD	
Year NAFLD diagnosed	
Investigations for NAFLD diagnosis	
Previous hepatology review	
Current Medications	
Lifestyle	
Smoking status – yes/no/previous and pack-years	
Alcohol consumption	
Physical activity	
Depression Inventory	
Family history of liver disease	
Fatty liver disease	
Liver disease	
Cirrhosis	
Liver cancer	

The patient will be diagnosed with NAFLD on the basis of the FibroScan CAP score (CAP score ≥ 248 dB considered as likely steatosis based on cut-offs determined in a large meta-analysis) [38] and stratified to low or high risk of clinically significant fibrosis on the basis of the FibroScan liver stiffness measurement (LSM).

No NAFLD: Participants with CAP score < 248 dB and LSM < 8 kPa will be classified as “no NAFLD”. A letter will be sent to the patient’s GP and diabetes specialist advising a repeat FibroScan or liver ultrasound in 3–5 years if appropriate, to screen for development of NAFLD. Participants will be informed of their FibroScan result at the NAFLD-RRR appointment and advised to follow up with their GP.

NAFLD with low risk of clinically significant fibrosis: Participants with CAP score ≥ 248 dB and low FibroScan score (LSM < 8.0 kPa) will be classified as ‘Low Risk’. A letter will be sent to the patient’s GP and diabetes specialist advising a repeat FibroScan in 2–3 years if appropriate, to assess for development of clinically significant fibrosis. If liver enzymes are abnormal, the letter to the participant’s GP will include guidance for further evaluation of abnormal liver enzymes.

High risk of clinically significant fibrosis: Participants with LSM ≥8.0 kPa will be classified as ‘High Risk’. The study clinician will advise the patient (and GP and diabetes specialist via letter) that Hepatology referral is recommended. A letter will be sent to the patient’s GP advising them to arrange a Hepatology Clinic referral. To assist GPs with this process, referral guidelines will be included with the patient’s letter and FibroScan result. If advanced fibrosis is confirmed in the secondary care hepatology clinic, the patient may be offered ongoing hepatology follow-up that may involve HCC and variceal surveillance programs.

For all patients with NAFLD, the letter to the GP and diabetes specialist will provide recommendations regarding management of NAFLD with ongoing assessment and management of cardiometabolic risk factors and lifestyle intervention with consideration of referral to a dietician, exercise physiologist and psychologist for assistance with weight management and increased physical activity. Patients diagnosed with NAFLD at the study clinic visit will receive a patient letter at the conclusion of the clinic visit. This letter advises patients to make an appointment to see their GP to further discuss the recommendations advised by the study team. The template letters are provided in Supplementary Figs. 1-4.

### Participant outcomes

#### Primary outcomes

- ***Detection and documentation of NAFLD in people with T2D.*** A diagnosis of NAFLD will be made following a participant’s FibroScan with a CAP score ≥ 248 dB, with documentation in a letter to the patient’s GP and Diabetes Specialist.
- ***Detection and documentation of significant liver fibrosis in people with T2D.*** This will be calculated based on the number of participants who receive a diagnosis of significant fibrosis (LSM ≥8.0 kPa), with documentation in a letter to the patient’s GP and Diabetes Specialist.

#### Secondary outcomes

- ***Rate of referral of “high-risk” NAFLD to Hepatology***. This will be calculated based on the number of “high-risk” patients referred to Hepatology clinics.
- ***Rate of referral of “low-risk” NAFLD to Hepatology.*** This will be calculated based on the number of participants who receive a diagnosis of NAFLD without clinically significant fibrosis (LSM < 8.0 kPa), and the number of these “low-risk” patients that are referred to Hepatology clinics.
- ***Rate of surveillance for HCC among “high-risk” patients.*** This will be calculated based on the number of participants with cirrhosis who are referred for surveillance for HCC according to guidelines (with liver imaging, usually ultrasound, every 6 months).
- ***Collection of evidence about the economic benefits of a NAFLD detection/risk stratification pathway in people with T2D.*** Costs associated with the new pathway will be collected by the research team using published costing methods. Modelling will permit estimation of an incremental cost per appropriate referral via the pathway versus usual care that can be considered in light of undesirable consequences, including those associated with delayed detection and referral. Diagnostic performance of the pathway in identifying cases of advanced fibrosis will be compared with usual care.
- ***Longitudinal outcome data.*** Participant outcome data will be collected at 12 months, 5 years and 10 years post recruitment. Linkage to Hospital Admitted Patient Data, Emergency Department data, Cancer Registry, and Death Registration will be performed by the Queensland Health Data Linkage Team for all participants. Data items will include: 1) details about hospital admissions and emergency presentations: dates, primary/other diagnosis, procedures and use of hospital-based allied health services and hospital-based clinical costings; 2) cancer (any type): date of diagnosis, site, morphology, differentiation, basis of diagnosis; 3) date and cause of death.
- ***Development of procedures and referral pathways to facilitate patient access to required services.*** The study clinician will develop resources for the service (GP template letters). Knowledge and attitudes of clinic/community clinicians will be surveyed post-implementation of the pathway (at 2 years).
- ***Collection of usual care data.*** Usual care data will be collected retrospectively from the three study sites for the year prior to recruitment commencement. Eligibility and exclusion criteria of controls will be the same as the intervention group except for patient consent (we will seek a waiver of consent to collect available data from patient medical records). The data collected for this usual care group will include demographic information, anthropometric measurements (height, weight, BMI, waist circumference), relevant metabolic co-morbidities, diabetes medications, and whether there is a record of alcohol consumption, a diagnosis of NAFLD and management of NAFLD – including risk stratification, blood tests, simple fibrosis scores, radiological investigations and/or referral to liver clinics.

### Statistical analysis

#### Sample size and statistical power

We aim to recruit at least 400 patients with T2D, anticipating ~ 60% to have NAFLD (*n* = 240). If 20% of these have advanced fibrosis [8] then the 95% confidence interval will be 15.4 to 25.5%. This will provide sufficient confidence that the observed rate using the pathway differs substantially from historical detection rates of < 5% [39].

#### Data analysis

Differences in the proportion of patients diagnosed with NAFLD (Aim 1), identified to have advanced fibrosis/cirrhosis (Aim 2), referred to Hepatology (Aim 3), and enrolled in a surveillance program for HCC (Aim 4) before and after implementation of the intervention will be assessed using statistical tests for categorical data (e.g. Pearson’s chi-squared test, McNemar test). Where appropriate we will use regression models to identify associations between variables (eg the association between treatment group (pathway vs. usual care) and clinical and sociodemographic factors).

#### Per protocol definition

We will use an intention-to-treat (ITT) approach meaning that participants will be analysed according to their recruitment, regardless of whether they followed the model of care [40].

In a sensitivity analysis we will use a per protocol (PP) analysis by only including patients that attended their FibroScan appointment and were able to be scanned. The per protocol analysis will be applied to the primary and secondary outcomes, and the economic analysis.

An intention-to-treat approach gives an indication of the value of the model of care in practice, because it includes the missed appointments that will happen in other clinics. A per protocol approach gives an indication of the potential benefits of the model of care. If the PP results show a larger benefit than the ITT results, then it provides impetus for finding ways to help patients attend their FibroScan appointment.

#### Missing data

We will report the number and percent of missing data for every study variable. For variables with relatively high levels of missing data (> 5%) we will investigate the reason for this and the potential for bias.

#### Resource use and costs

Healthcare resource use information will be collected for each of the pathways (NAFLD-RRR pathway and usual care). We will collect healthcare resource use information from two sources with equivalent availability for each group: administrative data collections from participating health services and auditing of clinical case note files. This will include capturing resource use related to the diagnosis of NAFLD, assessment of fibrosis, procedures and tests. Healthcare resource use will be costed from the healthcare funder perspective using actual costs (when available), market rates or published pricing information (where relevant).

#### Evaluation of knowledge and attitudes of clinic/community clinicians

A mixed-methods evaluation approach will be used, including the collection of quantitative data through participant surveys and qualitative data through participant interviews and open-ended survey questions.

## Discussion

Our health system research study examines a community-based NAFLD diagnosis, risk-stratification, and referral pathway for people with T2D. Compared with the general population, people with T2D have a higher prevalence of NAFLD and are at increased risk of developing the more severe inflammatory disease NASH, progressive liver fibrosis, and HCC [10, 41]. Targeting this at-risk population and providing a clear pathway linking primary care and liver clinics will lead to earlier identification and appropriate management of people with a greater prospect of adverse liver-related outcomes.

Since advanced fibrosis (F3/F4) is the key prognostic factor in NAFLD [18], a noninvasive point of care test to reliably identify this subgroup is crucial. Previous studies suggest that simple fibrosis scores perform less well in people with diabetes and have a lower predictive value to detect NAFLD-related advanced fibrosis [42, 43]. In this study the direct use of liver FibroScan provides a well-validated real-time assessment of liver fibrosis as well as steatosis, and allows for timely delivery of the diagnosis and fibrosis risk category (low or increased risk) to the patient’s healthcare team.

An important component of this study is the provision of a letter to the patient’s GP and diabetes clinician that provides succinct guidance on the appropriate management and follow-up of their patient in primary care, as well as investigation of abnormal liver enzymes or referral to a liver clinic if required. This “case-based” learning approach supports primary care clinicians to manage NAFLD in their community practice, as primary care is where most of the healthcare in Australia is coordinated. The letters will be evaluated for their effectiveness in providing guidance to deliver evidence-based care for people with this chronic health condition. Additional objectives include improving the GP’s capacity to recognize, diagnose, risk stratify and manage other people with NAFLD in their practice population, and to foster collaboration and integrated care with colleagues to improve their patients’ metabolic health.

Strengths of this study include the in-depth phenotyping of patients which will enable a thorough analysis of health outcomes and confounding factors to be performed longitudinally. Key outcomes include major adverse cardiac events (inclusive of myocardial infarction, cerebrovascular accidents, or hospitalisation for heart failure), extrahepatic malignancy, development of chronic kidney disease (decrease in glomerular filtration rate < 60 mL/min/1.73m^2^ for ≥3 months or markers of kidney damage) [44], hepatic-related events, and death. The predictive power of LSM and CAP for hepatic and extra-hepatic outcomes currently has sparse evidence, especially in the high-risk type 2 diabetes population.

Most studies have focused on clinical utility of FibroScan for the detection and risk stratification of NAFLD but there is limited real-world implementation research on the barriers to utilising current non-invasive tests (NITs) which will be assessed through focus groups and questionnaires. This knowledge gap was recognised in the consensus recommendations from the NAFLD public health agenda published this year [45]. There was unanimous agreement from the consensus group that there are “both economic and social arguments for taking action on NAFLD” and “early intervention could help reduce the burden of disease, associated health-care costs and economic losses” [45]. While there is data [46, 47] to support the economic burden of NAFLD with advanced chronic liver disease there is a lack of health economic data on implementing a screening program to detect early disease. In the NAFLD-RRR study we will be able to calculate the real-world costs of implementing a local screening program in a population at high-risk for advanced chronic liver disease.

This study does have limitations. The sole use of FibroScan for a diagnosis of advanced chronic liver disease is likely to overestimate the prevalence of advanced disease owing to its modest positive predictive value of 59% for advanced fibrosis [23]. However the diagnostic gold standard of liver biopsy carries an unjustifiable risk of adverse outcomes if implemented as a screening tool. FibroScan is a well validated NIT that can simultaneously assess hepatic steatosis and the risk of advanced fibrosis as a “point-of-care” test. It has one of the highest negative predictive values (84%) of all NITs currently available for clinical use in Australia to exclude advanced fibrosis/cirrhosis in people with type 2 diabetes [23]. Although the positive predictive value is lower at 59%, the reporting of outcome measures in this population will reduce the clinical significance of this overestimation [23]. Whilst allowing a rapid assessment for NAFLD, FibroScan requires a dedicated operator and is not currently widely available in the community. ShearWave elastography, available in many community radiology centres, has similar accuracy to FibroScan for the exclusion of advanced fibrosis although the test is not as well validated as FibroScan [48, 49].

Our study will provide important information about the feasibility of establishing a NAFLD pathway for people with T2D in primary care and the costs and health outcomes associated with the pathway compared to those experienced under usual care. The study represents a change to the current management model for chronic liver disease, in which people seek care to treat complications of progressive fibrosis, rather than maintaining health through illness prevention. As recently reported by the President of the American Academy of Family Physicians “ … we need to invest in a new model that would allow primary care clinicians and their teams to coordinate care locally, collaborate with community organizations and public health departments, maximize strengths of specialists, and address known social drivers of health … ” [50, 51].

## Supplementary Information

**Additional file 1: Supplementary Figure 1.** Participant Letter if NAFLD Identified.

**Additional file 2: Supplementary Figure 2.** Informative letters for GPs for participants classified as low risk.

**Additional file 3: Supplementary Figure 3.** Informative letters for GPs for participants classified as low risk with abnormal liver enzymes.

**Additional file 4: Supplementary Figure 4.** Informative letters for GPs for participants classified as high risk.

## Data Availability

The materials supporting the study methods are available within the article and its supplementary materials. Data sharing is not applicable as the manuscript does not contain any data.

## Abbreviations

NAFLD: Non-alcoholic fatty liver disease
HCC: Hepatocellular carcinoma
LFTs: Liver function tests
T2D: Type 2 diabetes mellitus
LSM: Liver stiffness measurement
CLD: Chronic liver disease
NFS: NAFLD Fibrosis Score
FIB-4: Fibrosis 4 Index
