# Supplementary figures and images for: Implementing the right care in the right place at the right time for non-alcoholic fatty liver disease (NAFLD-RRR study): a study protocol for a community care pathway for people with type 2 diabetes
Source: BMC Health Serv Res. 2022 Apr 12;22:487. doi: 10.1186/s12913-022-07808-7 (PMC9004198; doi:10.1186/s12913-022-07808-7)

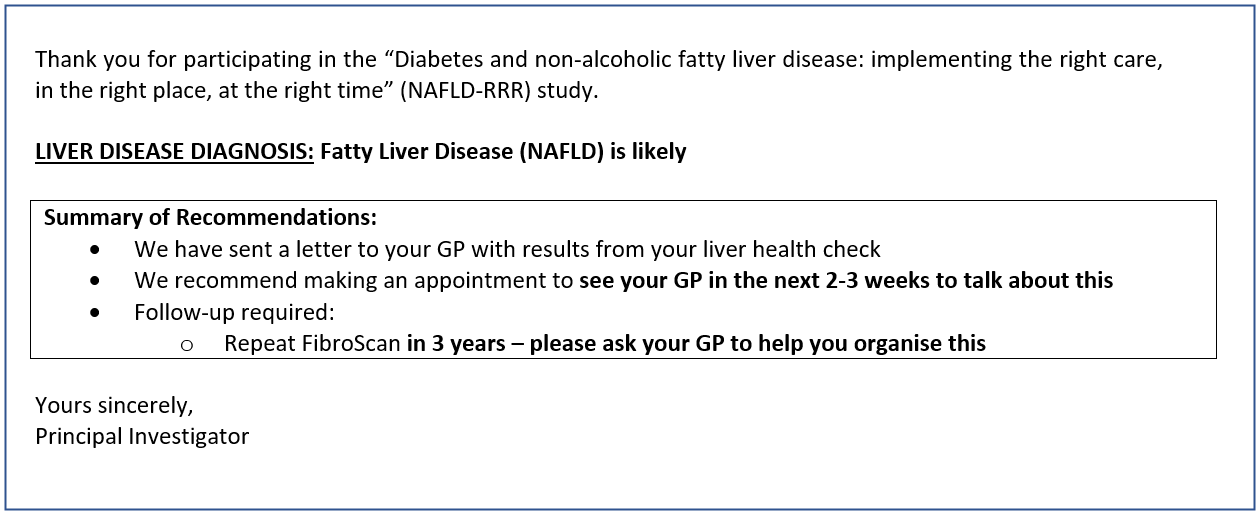

Supplement: Supplementary file 1 — Additional file 1: Supplementary Figure 1. Participant Letter if NAFLD Identified. [file 12913_2022_7808_MOESM1_ESM.png]

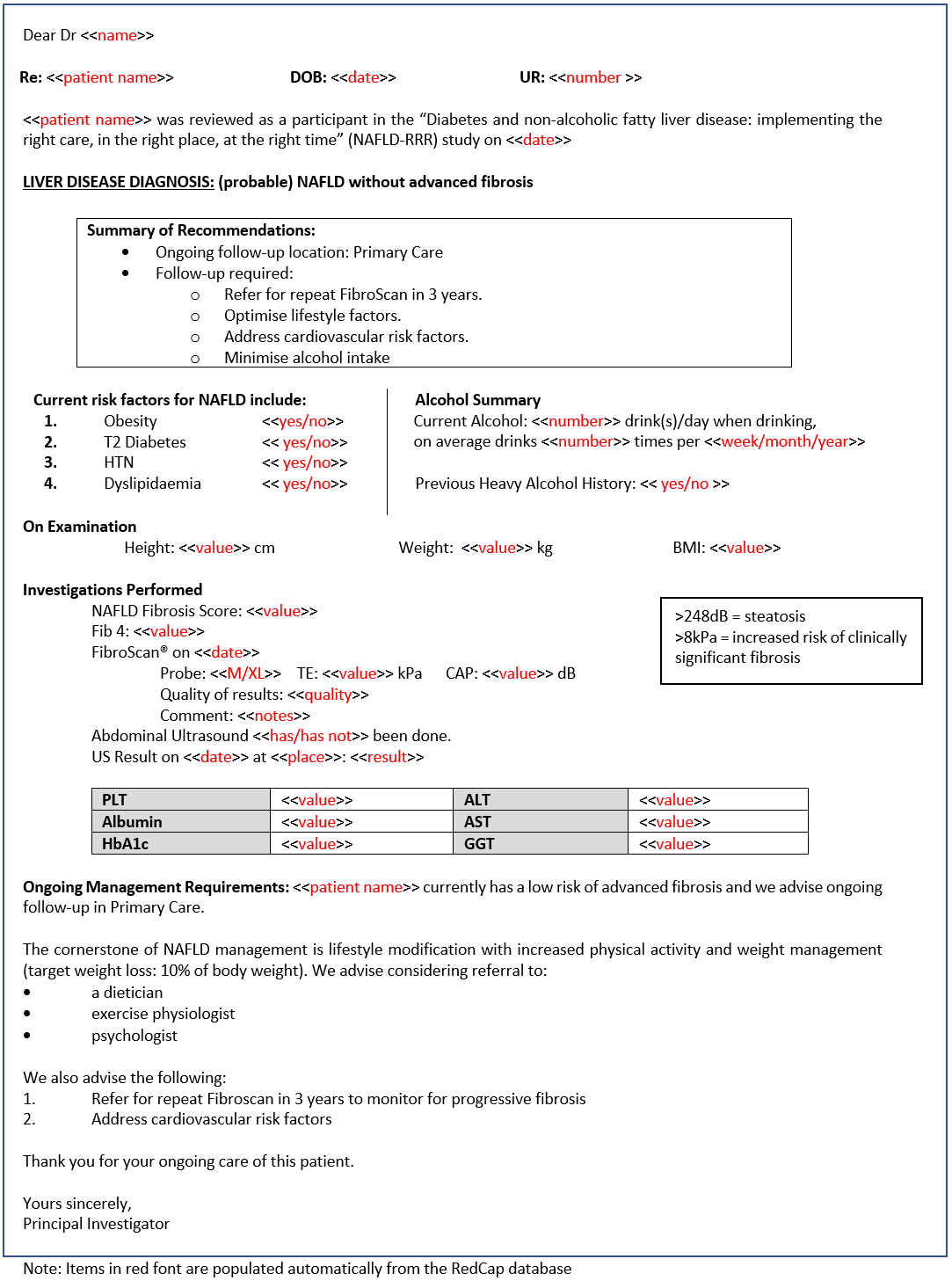

Supplement: Supplementary file 2 — Additional file 2: Supplementary Figure 2. Informative letters for GPs for participants classified as low risk. [file 12913_2022_7808_MOESM2_ESM.png]

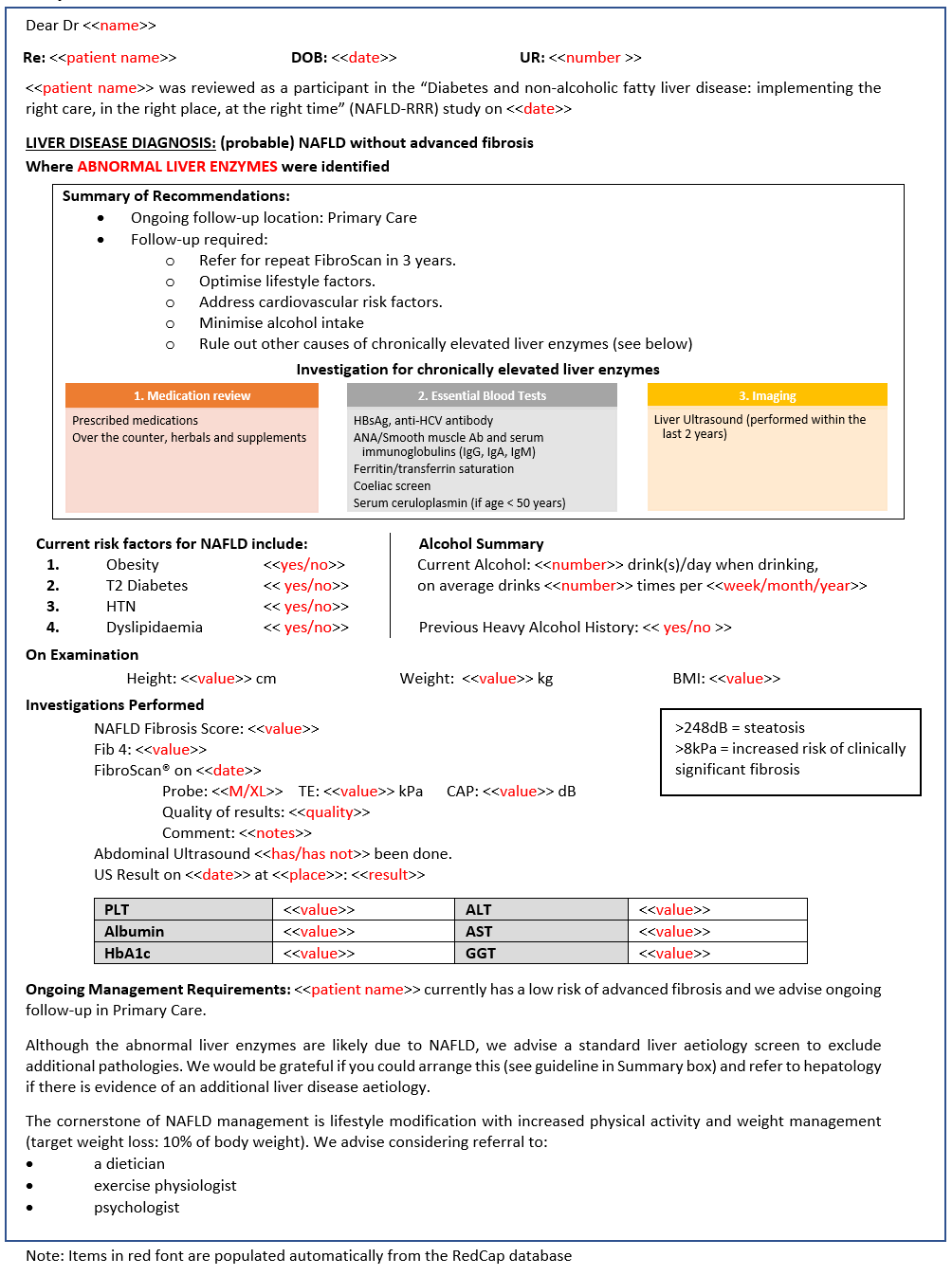

Supplement: Supplementary file 3 — Additional file 3: Supplementary Figure 3. Informative letters for GPs for participants classified as low risk with abnormal liver enzymes. [file 12913_2022_7808_MOESM3_ESM.zip › Supplementary Figure 3AR0.png]

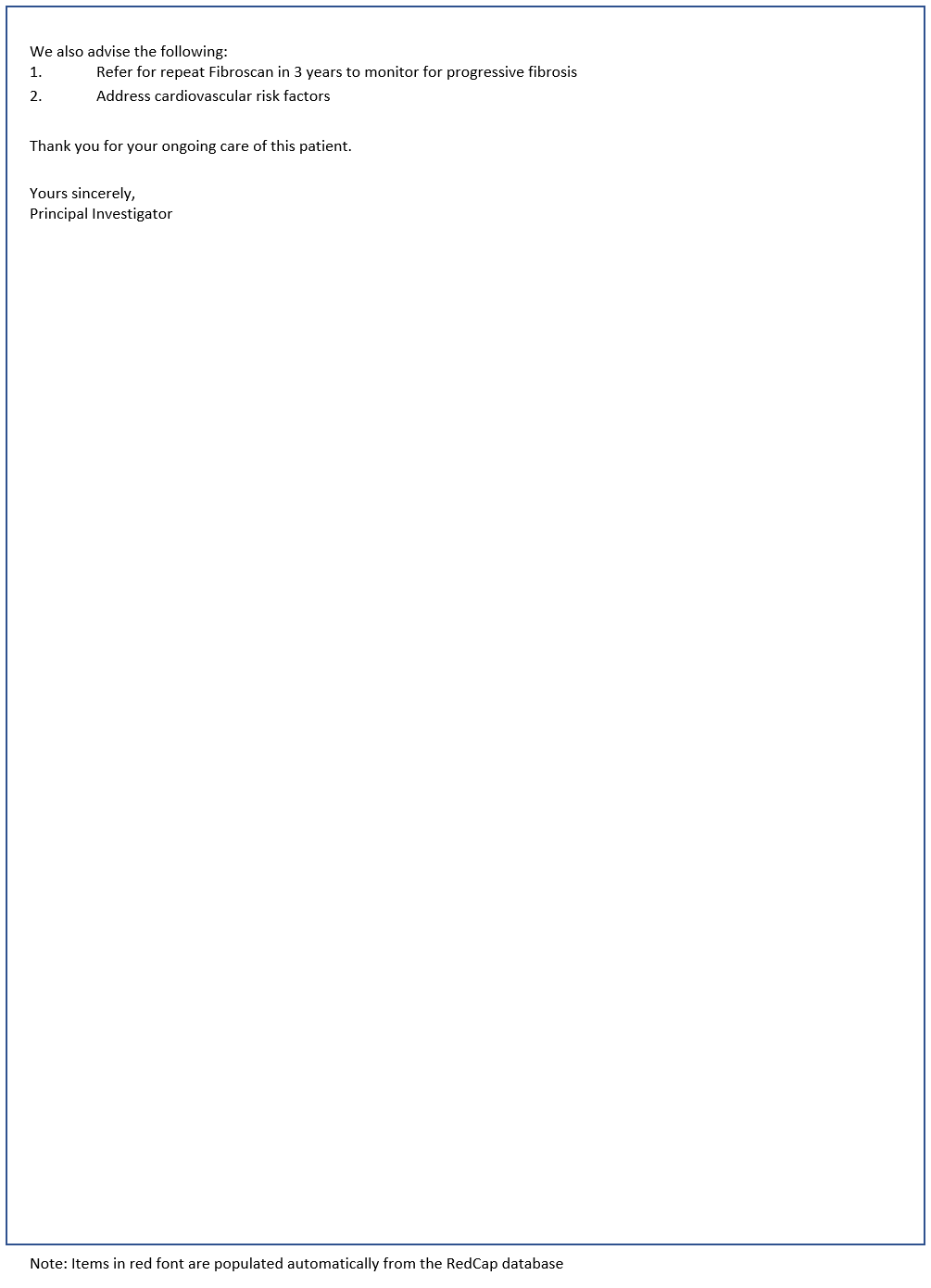

Supplement: Supplementary file 3 — Additional file 3: Supplementary Figure 3. Informative letters for GPs for participants classified as low risk with abnormal liver enzymes. [file 12913_2022_7808_MOESM3_ESM.zip › Supplementary Figure 3BR0.png]

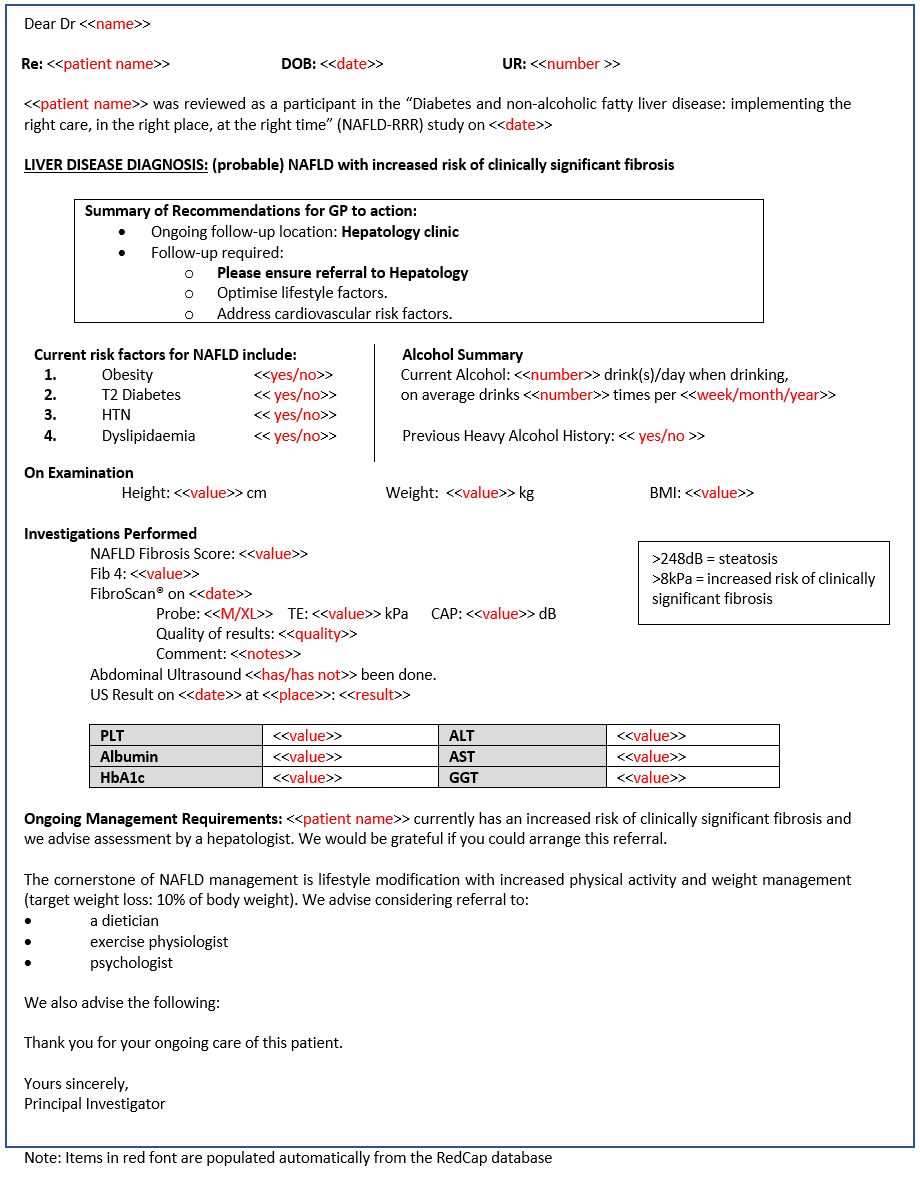

Supplement: Supplementary file 4 — Additional file 4: Supplementary Figure 4. Informative letters for GPs for participants classified as high risk. [file 12913_2022_7808_MOESM4_ESM.png]
